# Neonatal Cardiovascular-Progenitor-Cell-Derived Extracellular Vesicles Activate YAP1 in Adult Cardiac Progenitor Cells

**DOI:** 10.3390/ijms24098088

**Published:** 2023-04-30

**Authors:** Lourdes Ceja, Sean S. Escopete, Lorelei Hughes, Larry V. Lopez, Victor Camberos, Paul Vallejos, Nathan R. Wall, Mary Kearns-Jonker

**Affiliations:** 1 Department of Pathology and Human Anatomy, Loma Linda University School of Medicine, Loma Linda, CA 92350, USA; 2 Division of Basic Sciences, Loma Linda University School of Medicine, Loma Linda, CA 92350, USA

**Keywords:** cardiac progenitor cells, neonates, Islet-1, extracellular vesicle, cardiac repair, miRNAs, proliferation, cell cycle, YAP1, stem cells

## Abstract

New stem cell and extracellular-vesicle-based therapies have the potential to improve outcomes for the increasing number of patients with heart failure. Since neonates have a significantly enhanced regenerative ability, we hypothesized that extracellular vesicles isolated from Islet-1+ expressing neonatal human cardiovascular progenitors (CPCs) will induce transcriptomic changes associated with improved regenerative capability when co-cultured with CPCs derived from adult humans. In order to test this hypothesis, we isolated extracellular vesicles from human neonatal Islet-1+ CPCs, analyzed the extracellular vesicle content using RNAseq, and treated adult CPCs with extracellular vesicles derived from neonatal CPCs to assess their functional effect. AKT, ERBB, and YAP1 transcripts were elevated in adult CPCs treated with neonatal CPC-derived extracellular vesicles. YAP1 is lost after the neonatal period but can stimulate cardiac regeneration. Our results demonstrate that YAP1 and additional transcripts associated with improved cardiovascular regeneration, as well as the activation of the cell cycle, can be achieved by the treatment of adult CPCs with neonatal CPC-derived extracellular vesicles. Progenitor cells derived from neonates secrete extracellular vesicles with the potential to stimulate and potentially improve functional effects in adult CPCs used for cardiovascular repair.

## 1. Introduction

Cardiac-progenitor-cell-derived extracellular vesicles represent a promising cell-free therapy for cardiac repair. CPC-derived extracellular vesicles reduce infarct size and improve cardiac function post-infarction [1]. Extracellular vesicles derived from cardiac progenitor cells expressing MEF2C, GATA4, and Mesp1 include exosomes which are cardioprotective and promote angiogenesis [2]. Functional differences in cardiac protection have been reported in CPC-derived exosomes when compared with bone-marrow-derived mesenchymal-stem/progenitor-cell-derived exosomes [2], suggesting that cardiac progenitor cells may represent an optimal source of extracellular vesicles for cardiac repair. Since the neonatal heart is capable of regeneration, which is rapidly lost after birth [3,4,5], the characterization of CPC-derived extracellular vesicles isolated from Islet-1+ neonatal CPCs would benefit regenerative medicine. We hypothesized that the co-culture of neonatal exosomes with adult CPCs may activate transcripts associated with survival and proliferation in recipient cells that would improve their currently limited ability to promote cardiovascular repair. The transcriptome of extracellular vesicles derived from Islet-1+ CPCs was selected for this study since Islet-1 is expressed in a population of self-renewable, multipotent cells which are present in human neonates and have the ability to differentiate into all cells of the cardiovascular lineage [6]. Furthermore, Islet-1 expression is required for normal cardiac development, and cells expressing Islet-1 facilitate cardiac repair [6,7]. In this report, we utilize transcriptomic data and functional analysis to provide further insight into the unique content of neonatal Islet-1+ CPC-derived extracellular vesicles and their role in facilitating cardiovascular regeneration.

## 2. Results

### 2.1. The Transcriptome of Islet-1+ Human Neonatal Cardiac-Progenitor-Cell-Derived Extracellular Vesicles

**Figure 1:**
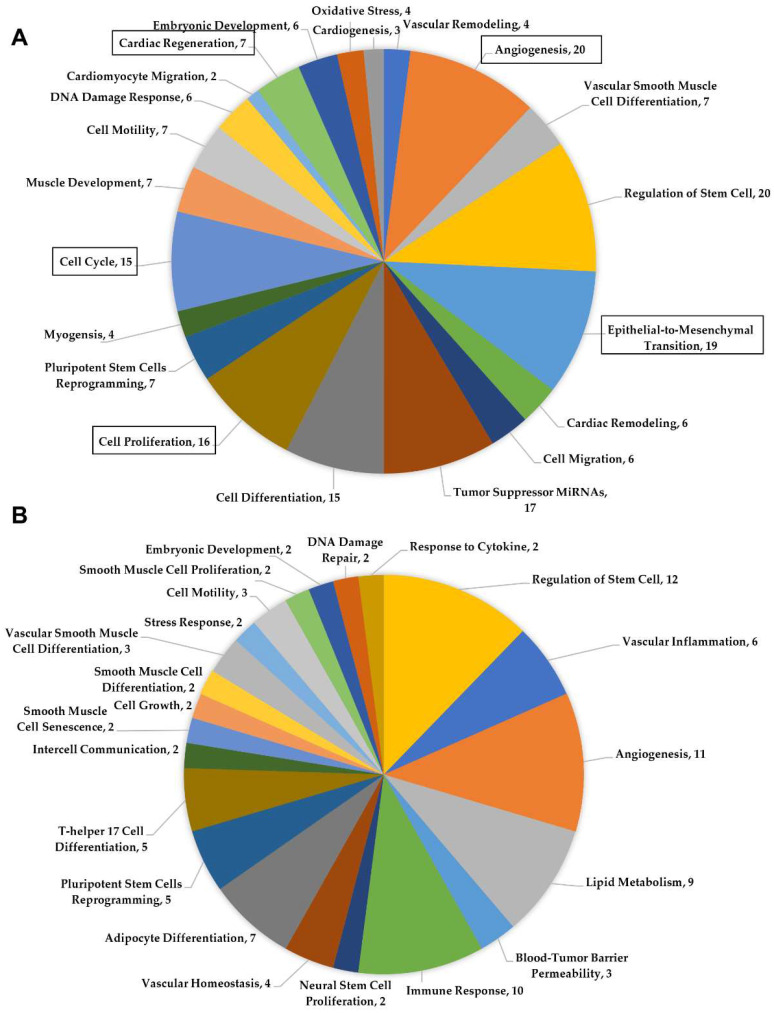
(**A**) Functional pathways identified by miRNet using miRNAs found within the neonatal cardiac-progenitor-cell-derived extracellular vesicle content. (**B**) miRNet analysis of miRNAs identified in the adult cardiac-progenitor-cell-derived extracellular vesicles.

The miRNA transcriptomic signature of human neonatal and adult cardiac-progenitor-cell-derived extracellular vesicles was compared using miRNet in order to identify functional differences associated with cardiac repair. We discovered several predicted functional pathways within the miRNA signature of neonates that differed from that of adults. Some of these included epithelial to mesenchymal transition, cell cycle, cell proliferation, and cardiac regeneration (Figure 1A). The neonatal extracellular vesicle content was predicted to impact the activation of the cell cycle and cell proliferation. Stimulation of the cell cycle and a substantial proportion of cell proliferation in the myocardium is necessary in order to successively replace the lost tissue following cardiac injury. miRNAs predicted to impact angiogenesis were also more highly represented in neonatal CPC-derived extracellular vesicles (Figure 1A). Several additional miRNAs within the neonatal CPC extracellular vesicle content were predicted to impact the functional outcome category of cardiac regeneration. This functional category was not represented in the transcriptome of adult CPC-derived extracellular vesicles (Figure 1B).

### 2.2. The Transcriptome of Islet-1+ Neonatal Cardiac-Progenitor-Cell-Derived Extracellular Vesicles Is Predicted to Activate YAP1 and the Cell Cycle

**Figure 2:**
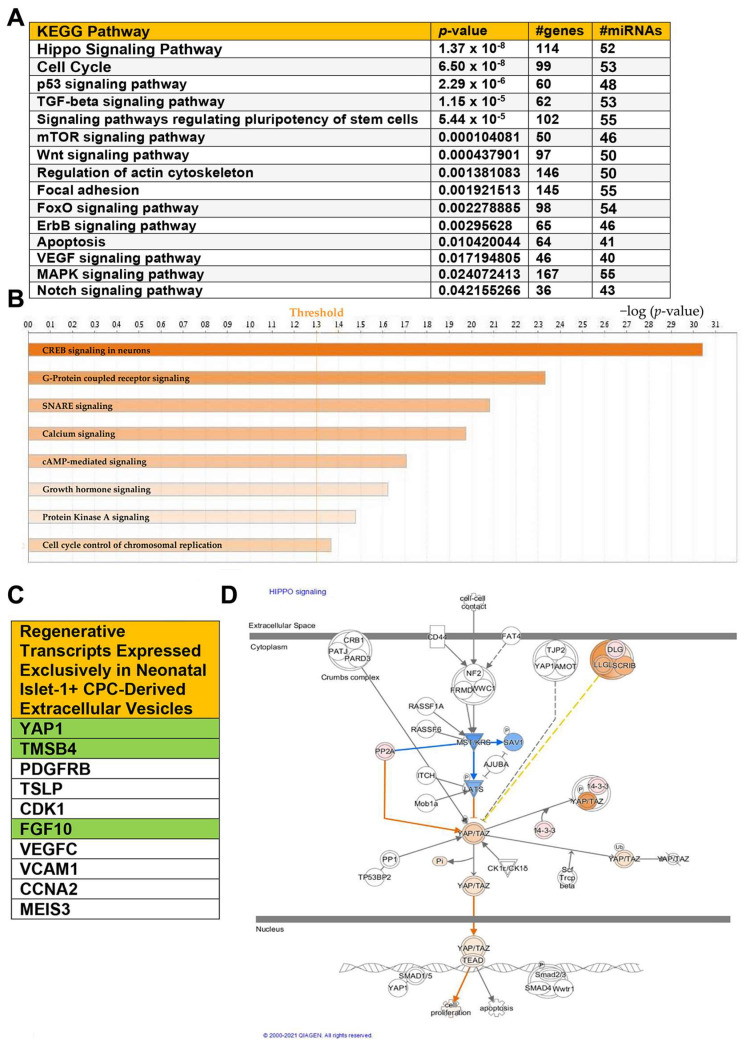
(**A**) KEGG analysis of miRNAs identified by RNA sequencing of Isl-1+ neonatal CPC-derived extracellular vesicles. (**B**) Canonical pathways significantly impacted by upregulated miRNAs and transcripts in the Isl-1+ neonatal CPC extracellular vesicle content. (**C**) Select transcripts associated with enhanced regenerative ability were identified in the neonatal Isl-1+ CPC extracellular vesicles but were not present in the extracellular vesicle content of the adult Isl-1+ CPC-derived extracellular vesicles. (**D**) The molecule activity predictor tool was used on IPA to predict the biological relationships between upregulated miRNAs and transcripts in relation to the Hippo signaling pathway. Orange color indicates predicted activation, while blue indicates predicted inhibition. A −log(*p*-value) of 1.3 is considered statistically significant.

The transcriptome of human neonatal Islet-1+ cardiac-progenitor-cell-derived extracellular vesicles was compared against the transcriptome of extracellular vesicles derived from human adult CPC clones. miRNAs which were differentially expressed, showing greater than a 2.0-fold change, were uploaded into DIANA-miRPath v3.0 bioinformatics software to identify which KEGG pathways were predicted to be regulated by the extracellular vesicle content of neonatal CPCs. Figure 2A identifies the pathways predicted to be regulated by miRNAs identified in neonatal CPC-derived extracellular vesicles. The most significant pathways found were the Hippo signaling pathway and cell cycle. Previous studies have shown that the Hippo signaling pathway is vital for cardiomyocyte proliferation in the postnatal heart through the activation of the downstream effector YAP1 [8]. As shown in Figure 2A, there are an estimated 114 genes targeted by about 52 miRNAs identified in the neonatal extracellular vesicle cargo regulating the Hippo signaling pathway. In addition to the Hippo signaling pathway, several other significantly impacted pathways listed in Figure 2A are also involved in the regeneration and development of the human heart. These pathways include cell cycle, Wnt signaling, ERBB signaling, and Notch signaling. 

DIANA miRPath is used to analyze miRNAs. We next uploaded transcripts showing greater than a 2.0-fold change for ingenuity pathway analysis. This was done to identify biological relationships between transcripts and miRNAs in the neonatal CPC extracellular vesicle content against the transcriptome of extracellular vesicles derived from adult CPCs. As shown in Figure 2B, several additional signaling pathways were identified as significantly impacted, including cyclic adenosine monophosphate response element-binding protein (CREB) signaling. CREB is able to promote the transcription of YAP1 by binding to the YAP1 promoter [9]. Several additional upstream components that have a significant impact on Hippo signaling include mechanical cues, G protein-coupled receptor signaling, and oxidative stress. Calcium signaling, cAMP-mediated signaling, growth hormone signaling, GP6, and protein kinase A signaling are all pathways critically necessary for physiological cardiac function and repair. 

Neonatal CPC-derived extracellular vesicles express numerous transcripts encoding proteins that contribute significantly to regeneration. Select transcripts were expressed exclusively in neonatal CPC-derived extracellular vesicles (Figure 2C). The transcripts highlighted in green are of special interest due to their previously documented role in the regeneration of the neonatal heart. Thymosin β4, for example, extends the regenerative window, while FGF10 and YAP1 are associated with the Hippo signaling pathway [10,11,12,13].

IPA allows for the identification of predicted molecule activity in both upstream and downstream regulation of signaling pathways. The molecule activity predictor (MAP) indicates that YAP1 is predicted to translocate into the nucleus and interact with transcriptional factors involved in the activation of cell proliferation (Figure 2D). 

### 2.3. Neonatal Cardiovascular-Progenitor-Cell-Derived Extracellular Vesicles Stimulate YAP1 Expression in Adult Cardiovascular Progenitor Cells

In order to examine the functional effects of neonatal CPC-derived extracellular vesicles on adult cardiovascular progenitor cells in vitro, we isolated Islet-1+ neonatal cardiac-progenitor-cell-derived extracellular vesicles and quantified them using Nanosight.

**Figure 3:**
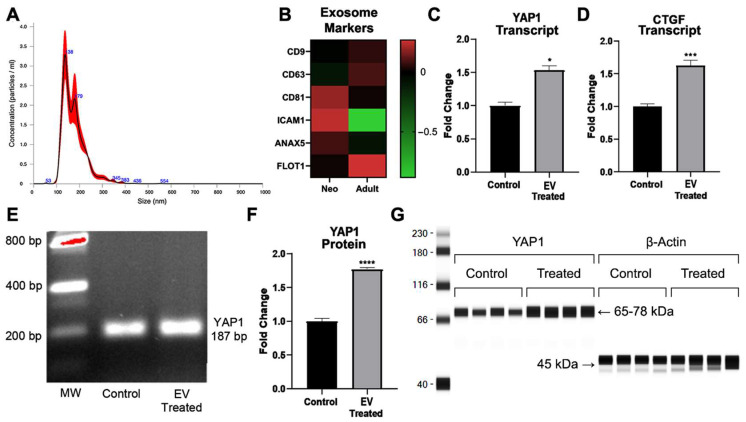
(**A**) Nanosight was used to identify the size of extracellular vesicles (EVs) isolated from neonatal Islet-1+ cardiac progenitor cell clones. The representative graph shows the size distribution profile data, n = 5, from one sample. Concentration measurements represent mean *±* standard error. (**B**) Common exosome markers were identified by transcriptomics in both neonatal- and adult-derived extracellular vesicle preparations. (**C**) YAP1 transcription was induced in the adult CPC following a 72 h treatment with extracellular vesicles isolated from Islet-1+ neonatal cardiovascular progenitor cells as shown by RT-qPCR. (**D**) CTGF, a well-documented downstream transcript which is elevated following intranuclear expression of YAP1, was increased in adult CPCs after co-culture with neonatal CPC-derived extracellular vesicles, as shown by RT-qPCR. (**E**) Gel electrophoresis was used to identify the correct size of the amplified RT-qPCR product YAP1 (187 bp). Quantification of protein levels was performed with the Protein Simple Wes automated gel-free Western blot system. (**F**) Analysis of YAP1 protein in the adult CPC following a 72 h treatment with extracellular vesicles isolated from Islet-1+ neonatal cardiovascular progenitor cells. (**G**) Corresponding visualization of YAP1 protein. YAP1 transcript and protein levels and CTGF transcripts were run in quadruplicate and normalized against the housekeeping gene β-Actin. Fold changes are shown with error bars indicating ± SEM. * indicates *p* ≤ 0.05, *** indicates *p* ≤ 0.001, **** indicates *p* ≤ 0.0001.

The ExoQuick-TC ULTRA-EV Isolation Kit resulted in the reproducible isolation of particles within the size range of <200 nm. The mean particle size was 176 nm, the concentration was 2.13 × 10^10^ ± 2.57 × 10^10^, and the mode was 137 nm, as shown in Figure 3A. Markers typically used to characterize exosomes were identified by transcriptomics in the isolated and sequenced extracellular vesicle preparations, as shown in Figure 3B. We next treated adult cardiovascular progenitors with extracellular vesicles derived from neonatal Islet-1+ cardiac progenitor cells in order to functionally assess YAP1 activation in the adult CPC. Adult cardiac progenitor cell clones received 5–6 × 10^10^ extracellular vesicles isolated from human neonatal Islet-1+ CPC clones or a similar volume of extracellular-vesicle-depleted media as a control. RNA and protein were extracted from adult cardiovascular progenitor cells after a 72 h treatment with neonatal Islet-1+ cardiovascular-progenitor-cell-derived extracellular vesicles. We assessed YAP1 and cell cycle transcripts and YAP1 protein levels in the adult CPCs after treatment. 

As can be seen in Figure 3C, statistical analysis revealed significantly elevated levels of YAP1 RNA (1.53 ± 0.25 FC, * *p* = 0.0286) after treatment with neonatal cardiovascular-cell-derived extracellular vesicles. In Figure 3E, we confirmed the transcript size of the RT-qPCR amplified product, YAP1, using gel electrophoresis (187 bp). Since we saw a significant increase in YAP1 transcripts after neonatal extracellular vesicle treatment, we quantified the protein levels of YAP1 in extracellular-vesicle-treated adult CPCs and the respective controls. We observed a significant increase in YAP1 protein (1.77 ± 0.09 FC, **** *p* < 0.0001) as a result of extracellular vesicle treatment when compared to the control, as shown in Figure 3F. The quantification and visualization of YAP1 protein is shown in Figure 3G. 

YAP1 activation leads to the transcription of downstream targets such as CTGF. We identified an increase in CTGF transcript levels (1.62 ± 0.33 FC, *** *p* < 0.0004) in adult CPCs after co-culture with neonatal CPC-derived extracellular vesicles, as shown in Figure 3D. The accumulation of YAP1 in the nucleus can lead to the activation of transcripts involved in proliferation. We therefore sought to identify other transcripts in the adult CPC that were impacted by neonatal CPC-derived extracellular vesicles and may contribute to YAP1 nuclear localization and proliferation. 

### 2.4. Neonatal Cardiovascular-Progenitor-Cell-Derived Extracellular Vesicle Co-Culture Stimulates the Expression of Prosurvival and Cell-Cycle-Associated Transcripts in Adult CPCs

#### 2.4.1. Transcripts Induced by Intranuclear YAP1 Activity

**Figure 4:**
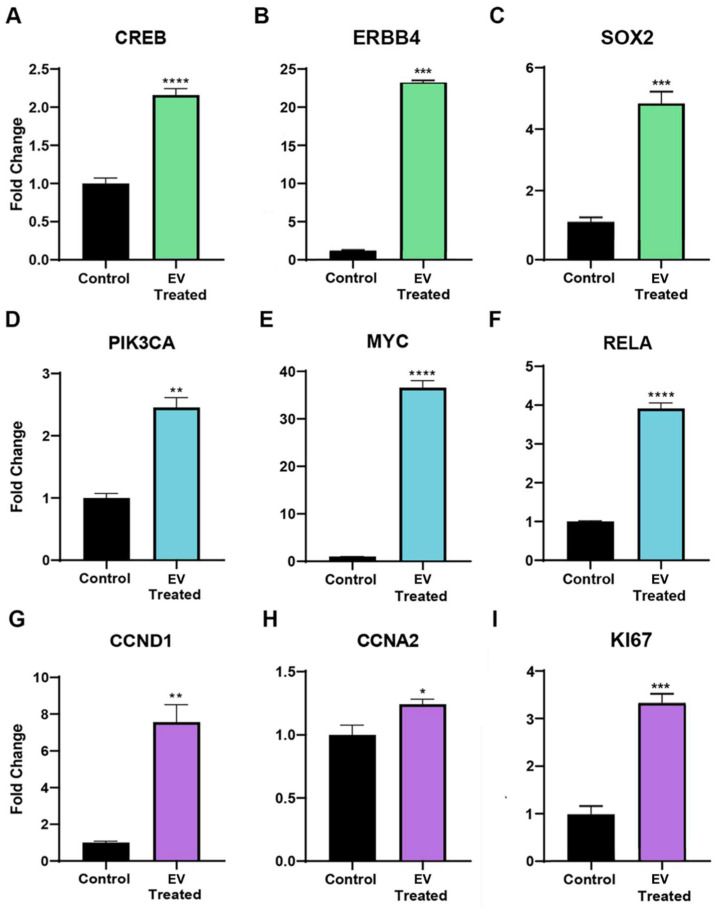
Transcripts associated with intranuclear YAP1, such as (**A**) CREB, (**B**) ERBB4, and (**C**) SOX2 transcripts, were found to be significantly elevated in adult CPCs after a 72 h neonatal CPC extracellular vesicle (EV) treatment, as shown by RT-qPCR. Quantitative RT-PCR data demonstrating transcripts associated with the AKT signaling pathway such as (**D**) PIK3CA, (**E**) MYC, and (**F**) RelA were found to be significantly elevated in the adult CPC following co-culture with extracellular vesicles isolated from Islet-1+ neonatal cardiovascular progenitor cells. Transcripts associated with the cell cycle and proliferation, such as (**G**) CCND1, (**H**) CCNA2, and (**I**) KI67, were significantly elevated in the adult CPC following a 72 h treatment with extracellular vesicles isolated from Islet-1+ neonatal cardiovascular progenitor cells, as confirmed by RT-qPCR. All samples were run in triplicate or quadruplicate and normalized to a housekeeping gene. Fold changes are shown with error bars indicating ± SEM. * indicates *p* ≤ 0.05, ** indicates *p* ≤ 0.01, *** indicates *p* ≤ 0.001, **** indicates *p* ≤ 0.0001.

YAP1 interacts with multiple pathways and can induce the transcription of genes involved in the promotion of proliferation, a process vital for organ growth. CREB signaling was found to be elevated in adult CPCs (2.16 ± 0.329 FC, **** *p* < 0.0001) after neonatal CPC extracellular vesicle treatment, as shown in Figure 4A. DNA-binding transcription factors such as ERBB4 also interact with YAP1 and are associated with proliferation. The YAP1-ERBB4 complex regulates organ and tissue growth. ERBB4 transcripts were found to be significantly increased in the adult CPC after extracellular vesicle treatment, as shown in Figure 4B. YAP1 interacts with ß-catenin to regulate the levels of SOX2, a transcript that supports cardiomyocyte proliferation. YAP1 induces SOX2 transcripts by occupying the promoter region of this gene. Co-culture of neonatal cardiac-progenitor-cell-derived extracellular vesicles with adult cardiac progenitor cells elevates SOX2 transcript levels, as shown in Figure 4C. Collectively, we identified several transcripts that are associated with the activation of YAP1, which were induced in adult cardiovascular progenitor cell clones following a 72 h exposure to neonatal cardiovascular-progenitor-cell-derived extracellular vesicles. We next addressed the influence of extracellular vesicles on the AKT signaling pathway due to the well-documented AKT/YAP1 crosstalk in proliferative, pro-survival networks [14].

#### 2.4.2. Transcripts Associated with AKT Signaling 

YAP1 nuclear localization and AKT signaling can induce proliferation [15] and CREB can activate the expression of YAP1 through AKT signaling. The AKT signaling pathway plays an important role in regeneration due to the influence of this pathway on cell cycle progression, cellular proliferation, motility, differentiation, angiogenesis, metabolism, and cellular survival. Transcripts associated with AKT signaling in adult CPCs were elevated, as demonstrated by RT-qPCR after treatment with extracellular vesicles derived from neonatal cardiovascular progenitors. These transcripts included PIK3CA. PIK3CA is able to activate AKT via the PI3K-AKT signaling pathway. PIK3CA expression was elevated 2.46 ± 0.47-fold, ** *p* = 0.0011 in adult CPCs after extracellular vesicle treatment, as shown in Figure 4D. The increased transcription of pro-proliferative genes in this pathway, such as MYC, occurs through the downstream targeting of PI3K-AKT and intranuclear YAP1 transcriptional activation. MYC positively regulates G1/S phase cell cycle progression by the regulation of cyclin E and CDK2 and, accordingly, MYC transcripts were elevated (36.59 ± 4.39 FC, **** *p* < 0.0001) in adult CPCs after co-culture with neonatal CPC extracellular vesicles, as shown in Figure 4E. AKT stimulates the transcription factor NF-κβ, a heterodimer composed of subunits p50 and RelA/p65. RelA/p65 can induce YAP1 nuclear localization and inhibit its degradation [16]. As a result of extracellular vesicle treatment, RelA transcripts were significantly increased (3.92 ± 0.42 FC, **** *p* < 0.0001) in adult CPCs, as shown in Figure 4F. 

#### 2.4.3. Cell Cycle Transcripts 

Both NF-κβ and YAP1 influence cell cycle control and proliferation by regulating the transcription of CCND1 [17,18]. As a result of neonatal Isl-1+ CPC-derived extracellular vesicle treatment, CCND1 transcripts were significantly increased in adult CPCs (7.56 ± 2.86 FC, ** *p* = 0.0024), as shown in Figure 4G. Intranuclear YAP1 translocation also upregulates CCNA2, a transcript present during the G_2_/M phase transition of the cell cycle. CCNA2 transcripts were elevated (1.24 ± 0.12 FC, * *p* = 0.0462) in the adult CPC following treatment with neonatal extracellular vesicles, as shown in Figure 4H. YAP1 activation in adult cardiomyocytes leads to the activation of pro-proliferative transcription factors such as KI67. KI67 mRNA is maximally expressed in G_2_ phase and mitosis, which was found to be significantly induced in the adult CPC after a 72 h co-culture with neonatal CPC extracellular vesicles, as shown by RT-qPCR in Figure 4I. Collectively, ERBB4, AKT-associated, and intranuclear YAP1-associated transcripts, which are elevated by extracellular vesicle treatment, lead to proliferation and cell cycle activity, as shown in Figure 4. 

#### 2.4.4. Proposed Signaling Mechanism by Which Adult Cardiac Progenitor Cells Are Induced to Proliferate In Vitro following Islet-1+ Neonatal CPC-Derived Extracellular Vesicle Treatment

It is known that extracellular vesicles can mediate downstream signaling events through ligand–receptor interactions or through the fusion and release of extracellular vesicle contents into the cytosol. This subsequently alters the physiological properties of the recipient cell [19]. In the context of influencing cellular proliferation, PI3K-AKT and Hippo signaling interaction has been illustrated mainly through receptor tyrosine kinases. Upon the stimulation of these ligand–receptor interactions, the phosphorylation of PIK3CA/p110α, PIK3CB/p110β, or p110δ/PIK3CD and a p85 regulatory subunit will drive the further activation of downstream signaling in the PI3K-AKT signaling pathway. Active AKT can also phosphorylate the IKK complex, which leads to the enhancement of NF-κβ transcriptional activity. NF-κβ contains a family of transcriptional factors that function in inflammation, immunity, cellular proliferation, differentiation, and survival [20]. Some of these downstream targets include MYC and CCND1 [21]. AKT can phosphorylate CREB, which leads to the transcription of downstream genes involved in cellular proliferation and survival, such as BCL-2, CCND1, and cyclin A (CCNA2) [22].

**Figure 5:**
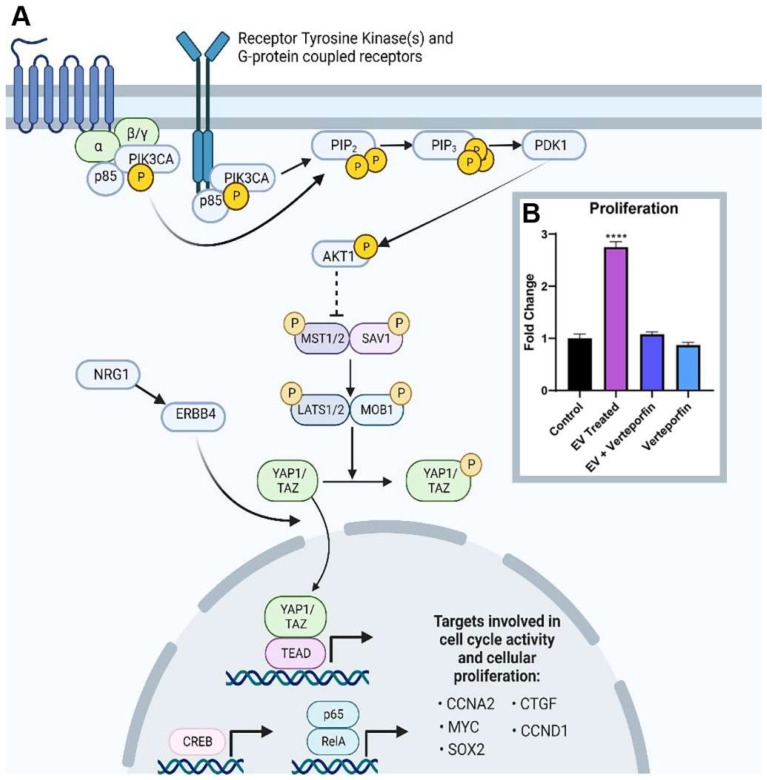
(**A**) Proposed signaling mechanism in adult CPCs upon co-culture of extracellular vesicles (EVs) isolated from neonatal Islet-1+ cardiac progenitor cells. Image created with BioRender.com. (**B**) Application of neonatal cardiovascular-progenitor-cell-derived extracellular-vesicles-induced proliferation in adult CPCs as measured by an in vitro proliferation assay. Verteporfin, a YAP1 inhibitor, blocked proliferation. There was no statistically significant difference between control, EV + verteporfin, and verteporfin alone. Fold changes are shown with error bars indicating ± SEM. **** indicates *p* ≤ 0.0001.

Activation of PI3K-AKT signaling occurs after extracellular vesicle treatment, associated with an increase in PIK3CA expression. AKT phosphorylates MST1/2, and the downstream effects lead to intranuclear YAP1 activity [23]. YAP1 transcripts and protein are elevated, leading to the transcription of downstream targets such as CTGF, SOX2, CCND1, and CCNA2. KI67 transcripts were also shown to be elevated. The proposed signaling mechanism induced in adult CPC by neonatal cardiovascular-progenitor-cell-derived extracellular vesicles is shown in Figure 5A. When adult and neonatal Islet-1+ CPC-derived extracellular vesicles were compared using transcriptomics, YAP1 was not identified in the adult extracellular vesicle transcriptome. MicroRNAs such as miR133a, which is capable of positively regulating YAP1, were included in the cargo carried by neonatal CPC-derived extracellular vesicles [Figure S1]. MicroRNA133 induces cardiomyocyte proliferation and enhances cardiac repair [24,25]. Following treatment with neonatal CPC-derived extracellular vesicles, proliferation was shown to be enhanced in adult CPCs in vitro (Figure 5B). To validate whether or not YAP1 was responsible for extracellular-vesicle-induced proliferation, cells were additionally treated with 3 µM of verteporfin, an established YAP1 inhibitor [26]. Verteporfin treatment blocked extracellular-vesicle-induced proliferation (Figure 5B), demonstrating that YAP1 was responsible for this effect. 

## 3. Discussion

Extracellular vesicles can be harnessed as a therapeutic approach for cardiac repair due to their natural ability to alter the transcriptome of recipient cells. We sought to examine the transcriptome of neonatal cardiac-progenitor-cell-derived extracellular vesicles in order to identify their content and provide insight into the pathways associated with regeneration, which are uniquely activated in neonates. 

The transcriptome of Islet-1+ neonatal CPC-derived extracellular vesicles may harbor the key to improving regenerative outcomes. We identified several thousand unique transcripts in the neonatal extracellular vesicle transcriptome when compared with the extracellular vesicle content of CPC derived from adults. According to IPA analysis, these transcripts can influence multiple pathways, including Wnt, ERBB, Notch, TGF-beta, Focal adhesion, CREB, G protein-coupled receptor signaling (GPCR), and Hippo. Transcripts and long non-coding RNAs, which are carried within the neonatal extracellular vesicle cargo, influence cardiomyocyte proliferation, oxidative stress, apoptosis, angiogenesis, and dedifferentiation. Especially promising is the impact of neonatal CPC-derived extracellular vesicles on the activation of YAP1 and the downstream transcripts induced by intranuclear YAP1 expression, which play a role in maintaining the expression of this pro-proliferative protein. YAP1 transcripts activate proliferation in fully differentiated surrounding cardiomyocytes [15,27] and can stimulate a pro-regenerative/pro-survival pathway in adult cardiovascular cell progenitors via the contribution of ERBB, IGF, and AKT signaling. 

We have demonstrated that neonatal CPC-derived extracellular vesicles activate transcripts induced by intranuclear YAP1 expression, including CTGF in adult CPCs. CTGF triggers cell cycle activity in neonatal mammals as well as cell migration and adhesion, angiogenesis, early wound healing, and repair [28,29,30]. Specifically, CTGF has been shown to provoke cell cycle activity by stimulating DNA synthesis in neonatal mammals [28]. ERBB4, another downstream transcript which is activated by intranuclear YAP1 expression, participates in cell-based repair by activating a YAP1 feedback loop [31]. YAP1 is downregulated after the neonatal period. The restoration of YAP1 activity in adult cardiomyocytes improves outcomes in cell-based repair. Several miRNAs identified within the neonatal cardiovascular-progenitor-cell-derived extracellular vesicle transcriptome facilitate this process, including miR-31, miR221, miR222, mir-181a/b, and miR133a. miR-31 and miR-221 are able to indirectly activate YAP1 transcriptional activity through the suppression of LATS [32,33]. In addition, miR-221 is capable of targeting cyclin-dependent kinase inhibitors (CDKI) CKKN1B/p27 and CDKN1C/p57. Consequently, miR-221 can promote proliferation through the modulation of cell-cycle-dependent genes [32]. The manipulation of the Hippo signaling pathway can influence the YAP1-mediated de-differentiation of adult cardiomyocytes [34]. De-differentiation is necessary for cell cycle re-entry and is a key step in activating cardiac regeneration [34]. The overexpression of miR-31 leads to YAP1 translocation, where it promotes the transcription of CCND1 and postnatal cardiomyocyte proliferation in vivo [29,33]. The upregulation of miRs-222 and miR296 similarly activate cardiomyocyte proliferation. 

Several additional signaling events relevant for cell-based repair are influenced by the content of neonatal CPC-derived extracellular vesicles. For example, Notch and Hippo signaling promotes the expression of neuregulin [35,36]. Neuregulin plays a role in ERBB signaling by interacting directly with ERBB2/ERBB4 receptors, stimulating cardiomyocyte proliferation [37]. Alterations in the actin cytoskeleton, TGF-beta signaling, and focal adhesion signaling can activate YAP1 transcriptional activity [38,39]. mTOR signaling, predicted to be activated by KEGG analysis, has an important role in the regulation of cellular growth, metabolism, proliferation, and survival [40,41]. Calcium signaling is required for early cardiac development and cardiomyocyte proliferation in the embryonic heart [42,43]. In addition, calcium signaling, paracrine communication, and transcriptional signaling are major processes involved in cardiac morphogenesis [42]. cAMP utilizes PKA to activate the epidermal growth factor signaling pathway and ERK1/2 signaling to activate cell proliferation [44]. Growth hormone signaling is necessary for liver regeneration [45], and SNARE signaling is involved in exosomal biogenesis and secretion [46]. Cell cycle control of chromosomal replication predicts that cyclin-dependent kinases are activated and transferred through extracellular vesicle platforms to nearby cells. This prediction can be an important mechanism for cardiac repair. 

In addition to modulating signaling pathways, the multi-faceted process of cell-based repair requires active cardioprotection from oxidative stress, apoptosis, and the inhibition of fibrosis. The neonatal extracellular vesicle cargo carries EREG and LIF as well as miRNA 370, which reduces oxidative stress and apoptosis. miR-133a, miR181a, and miR222 enhance the protective capacity of cardiovascular progenitors post-infarction by targeting genes related to cell death, fibrosis, and apoptosis [24]. The overexpression of miR-133a augments survival and regenerative capacity by elevating FGF and VEGF expression [24]. Paracrine effects promoted by neonatal extracellular vesicles also include the pro-proliferative and regenerative IGF2, HGF, and PDGFRB, all of which are widely acknowledged as contributors to cardiac regeneration [47,48,49]. 

The neonatal CPC extracellular vesicle cargo includes both unique transcripts, as well as transcripts that are expressed in extracellular vesicles derived from neonatal and adult cardiovascular progenitor cells. Numerous transcripts were expressed at significantly lower levels in adult CPC-derived extracellular vesicles. Expression levels have the potential to play a significant role in gene regulation. The overexpression of transcripts introduced into stem cells by methods such as viral transduction has provided evidence for the role of these genes in cardiac repair or regeneration. Several of these transcripts are naturally expressed at relatively high levels within the neonatal CPC extracellular vesicle content and include FGF1, FGF10, FGF11, and FGF17. Additionally, CPC-derived or MSC-derived extracellular vesicles transduced with chemokine receptor 4 (CXCR4) have been reported to significantly reduce infarct size and improve the left ventricular ejection fraction (LVEF) [50,51]. CXCR4 has implications in many regenerative-like processes such as cell mobilization, migration, proliferation, and survival [52]. In our study, neonatal cardiovascular progenitor cell extracellular vesicle fractions expressed CXCR4 transcripts at levels which were 7.71-fold higher than those expressed in adult CPC-derived extracellular vesicles. 

While much of the literature has focused on the beneficial effects of activating surrounding cardiomyocytes and reducing fibrosis, we provide additional information supporting the approach of activating stem cells obtained from older adult patients using neonatal stem-cell-derived extracellular vesicles to improve outcomes in cell-based repair. The activation of YAP1 signaling in adult stem cells promotes expansion, survival, retention, and augments the number of stem cells producing extracellular vesicles locally. These secreted extracellular vesicles have beneficial effects that include reducing inflammation of the surrounding tissue, supporting angiogenesis, and reducing oxidative stress. Our studies provide transcriptomic information defining the extracellular vesicle content of Islet-1+ neonatal CPC and identifying the mechanistic basis by which YAP1 is induced in adult CPC without the need for virally mediated transduction. The activation of adult cardiovascular progenitors with extracellular-vesicle-based pre-treatment alters the transcriptome to promote survival and enhance proliferation. Cell-free therapeutics using extracellular vesicles derived from cells with an enhanced regenerative ability may therefore be able to achieve improved outcomes when applied in vivo for cardiac repair. 

## 4. Materials and Methods

### 4.1. Isolation and Culture of Human Neonatal and Adult Islet-1+ Cardiovascular Progenitor Cells

The Institutional Review Board of Loma Linda University approved the protocol for the use of tissue that was discarded during cardiovascular surgery, without identifiable patient information, for this study with a waiver of informed consent. Human neonatal and adult Islet-1+ cardiac progenitor cell clones were previously isolated from discarded surgical cardiovascular tissue [53] and were available for use in this study. Briefly, discarded atrial tissue from human neonates and adults was digested in a collagenase solution (Roche Applied Science, Indianapolis, IN, USA), and clonal cell populations were established by limiting dilution. Human CPC clones used in the current project were cultured in growth media that included 10% fetal bovine serum (FBS) or extracellular-vesicle-depleted 10% FBS (Genesee Scientific, San Diego, CA, USA), medium 199 (Thermo Fisher Scientific, Waltham, MA, USA), 100 units/mL of penicillin–streptomycin (Gibco, Thermo Fisher Scientific, Waltham, MA, USA), 22% endothelial cell growth medium-2 BulletKit (Lonza, Basel, Switzerland), and 1% minimum essential medium non-essential amino acids solution (Life Technologies by Thermo Fisher Scientific, Waltham, MA, USA). 

### 4.2. Preparation of Extracellular Vesicle Depleted Fetal Bovine Serum

Extracellular vesicles were removed from the serum used for cell culture, incorporating an approach that removes approximately 95% of FBS-derived extracellular vesicles [54,55]. Briefly, fetal bovine serum (Genesee Scientific, San Diego, CA, USA) was subjected to ultracentrifugation at 100,000× *g* for 18 h at 4 °C. The supernatant was filtered with a 0.22 μm filter to reduce contamination and was stored at −18 °C until needed.

### 4.3. Collection, Isolation, and Quantification of Extracellular Vesicles from Islet-1+ Cardiovascular-Progenitor-Cell-Conditioned Media

Islet-1+ cardiovascular progenitor cells were cultured in 6-well 0.1% gelatin-coated plates with extracellular-vesicle-depleted 10% FBS-prepared medium. Cells were incubated at 37 °C with 5% CO_2_ and 95% oxygen until 90% confluency in order to isolate extracellular vesicles from the conditioned media. The extracellular-vesicle-containing conditioned media was saved at −80 °C and thawed at room temperature prior to extracellular vesicle isolation [56,57]. 

### 4.4. Exo-Quick-TC ULTRA EV Isolation Kit for Cell Culture Media

The Exo-Quick TC ULTRA EV isolation kit was used to purify extracellular vesicles from the media of cardiovascular progenitors according to the manufacturer’s instructions (System Biosciences, Palo Alto, CA, USA). Briefly, conditioned media was centrifuged at 3000× *g* in order to remove cellular debris. The supernatant was resuspended with the ExoQuick-TC reagent using a ratio of 5 mL of conditioned media to 1mL of reagent and was incubated overnight at 4 °C. Subsequently, the solution was centrifuged for 10 min at a speed of 3000× *g* at room temperature. The supernatant was aspirated to leave the precipitated extracellular vesicles for further collection. The pellet was resuspended with 200 μL of Buffer B, followed by the addition of 200 μL of Buffer A. The solution was loaded onto a prepared isolation column and placed on a rotating shaker for 5 min at room temperature. The purified extracellular vesicles were eluted at a speed of 1000× *g* for 30 s, and this step was repeated. A dilution of 3:1000 was prepared for Nanosight preparation and saved in −20 °C or −80 °C. 

### 4.5. Nanoparticle Tracking Analysis 

Analyses of particle size and concentration of extracellular vesicles isolated from cardiovascular-progenitor-cell-conditioned media was performed using a Nanosight NS300 instrument (Malvern Panalytical, Malvern, UK) and nanoparticle tracking analysis (NTA) software (version 3.4; Malvern Panalytical, Malvern, UK). Extracellular vesicle samples diluted in PBS (3:1000) were thawed at room temperature. Samples were vortexed and sonicated for 30–45 s before injection. Five videos of each sample determined the mean size and concentration of particles: flow rate, 30. 

### 4.6. RNA Sequencing and Transcriptomic Analysis of Adult and Neonatal Islet-1+ CPC-Derived Extracellular Vesicles

RNA samples were extracted and purified from the extracellular vesicles derived from adult and neonatal Islet-1+ cardiovascular progenitors. RNA samples were sent to the PrimBio Research Institute (Exton, PA, USA) for transcriptome analysis [58]. Briefly, rRNA was removed from total RNA samples with a rRNA removal kit from Illumina (San Diego, CA, USA). Ion Total RNA-Seq Kit v2 (Thermo Fisher Scientific, Waltham, MA, USA) was used to assemble sequencing libraries. Prior to PCR amplification, nucleic acid binding beads (Ambion, Austin, TX, USA) were used to purify the cDNA library. An agilent dsDNA High Sensitivity kit (Agilent, Santa Clara, CA, USA) was used to test the quality of libraries. Samples were enriched with an Ion OneTouch ED instrument and an Ion PI™ Hi-Q™ OT2 200 Kit (Thermo Fisher Scientific, Waltham, MA, USA). Sequencing was performed with an Ion Proton sequencer (Thermo Fisher Scientific, Waltham, MA, USA) and a species-specific protocol. Sequence files were aligned to the human genome, and quality of sequence files was assessed using the Strand NGS program. Quantification and normalization of aligned reads were performed using the DEseq algorithm within the strand NGS program. The Audic–Claverie test and the Benjamini–Hochberg correction test were used for statistical analysis. Significance was determined using a 2.0-fold change minimum cutoff. 

miRNAs, which were predicted as upregulated or downregulated and showing greater than a 2.0-fold change in neonatal CPC-derived extracellular vesicles when compared to adult CPC-derived extracellular vesicles, were uploaded to DIANA-miRPath v3.0 bioinformatics software [59]. A KEGG analysis was performed on the dataset based on the predicted targets of uploaded miRNAs. Categories specific to pathways involving development, proliferation, cell cycle, or the Hippo signaling pathway, and only categories with a *p*-value of < 0.05, were reported. Total gene transcripts that were predicted to be upregulated and showing greater than a 2.0-fold change were then uploaded for ingenuity pathway analysis (IPA) (Qiagen, Valencia, CA, USA). A core analysis was then performed. Categories specific to pathways involving exosome biogenesis, development, proliferation, cell cycle, or the Hippo signaling pathway and with a *p*-value of < 0.05 were reported. 

### 4.7. Treatment of Human Adult Cardiac Progenitor Cells with Extracellular Vesicles Isolated from Neonatal Cardiovascular Progenitors 

Adult CPC clones received 5–6 × 10^10^ extracellular vesicles which were isolated from human neonatal Islet-1+ CPC or a similar volume of extracellular-vesicle-depleted media as a control. Cells underwent a 72 h incubation at 37 °C with 5% CO_2_ and 95% oxygen before subsequent experiments were performed. 

### 4.8. Purification of RNA and Reverse Transcriptase Quantitative PCR

Adult treated and non-treated CPCs received 700 μL of QIAzol^®^ reagent (Qiagen, Valencia, CA, USA). Total RNA was purified using an RNeasy mini kit (Qiagen, Valencia, CA, USA) following the manufacturer’s instructions. A total of 1 μg of RNA was used to prepare cDNA using Superscript III (Invitrogen by Thermo Fisher Scientific, Waltham, MA, USA). Reverse transcriptase quantitative PCR was performed using iTaq™ Universal SYBR^®^ Green Supermix (Bi-Rad, Hercules, CA, USA). A Bio-Rad CFX96 Touch Real-Time PCR Detection System was used to perform all RT-qPCR experiments (Bi-Rad, Hercules, CA, USA). PCR plates were run with the following settings: 94 °C for 10 min, 94 °C for 15 s, 56–58 °C (depending on the primer) for 60 s, and 72 °C for 30 s, repeated for 45 cycles. Primers of our genes of interest were constructed using National Center for Biotechnology Information (NCBI) Primer-BLAST and are shown in Table 1. Reverse transcriptase quantitative PCR products were visualized using a 1–2% agarose gel electrophoresis and a low-mass DNA ladder to ensure that the correctly sized transcripts were amplified (Invitrogen by Thermo Fisher Scientific, Waltham, MA, USA). 

### 4.9. Protein Purification from Human Adult CPCs after Extracellular Vesicle Treatment for Western Blot 

Following a 72 h neonatal Islet-1+ CPC-derived extracellular vesicle treatment or control treatment, the adult CPCs were aspirated to remove any medium, washed with cold PBS (Genesee Scientific, San Diego, CA, USA), and were incubated with cold trypsin (Thermo Fisher Scientific, Waltham, MA, USA). Trypsinized cells were placed on ice until all the cells became detached. Adult extracellular vesicle and control-media-treated samples were resuspended in a protein lysis buffer solution consisting of RIPA buffer, 0.5M of EDTA, protease inhibitor cocktail, sodium orthovanadate, and sodium fluoride and were agitated for 1 h at 4 °C. The samples were then subjected to centrifugation at 14,000× *g* and aliquoted for use. The protein concentration was quantified using the Micro BCA Protein Assay Kit (ThermoFisher Scientific, Waltham, MA, USA). Simple Wes by Protein Simple (ProteinSimple, San Jose, CA, USA) is an automated gel-free Western blotting system and was used following the manufacturer’s instructions to analyze protein levels in adult CPCs. Antibodies used for Western blot are shown in Table 2 (Cell Signaling Technology, Danvers, MA, USA). 

### 4.10. Proliferation Assay 

Cells were seeded at a density of 2000 cells/well in a 96-well plate with 100 µL of cardiac progenitor cell media for 4 h until attachment was observed. Media was aspirated and replaced with 100 µL of extracellular-vesicle-depleted CPC growth media (EDM) as a control. EDM was generated via ultracentrifugation of CPC growth media at 100,000× *g* for 18 h at 4 C. The supernatant was filtered with a 0.22 m filter to reduce contamination and stored at 4 °C. The three treatment groups included 3.0 × 10^8^ extracellular vesicles suspended in 100 µL of EDM, 3 µM of verteporfin (Tocris, Bristol, UK) in 100 µL of EDM, and 3 µM of verteporfin and 3.0 × 10^8^ extracellular vesicles in 100 µL of EDM. Additional wells containing 100 µL of EDM without cells were added as an additional negative/blank control. Cells and controls were incubated for 72 h at 37 °C with 5% CO_2_ and 95% oxygen. The Quick Cell Proliferation Assay Kit Plus was used according to manufacturer’s instructions to quantify cellular proliferation (BioVision, Milpitas, CA, USA). Briefly, after a 72 h incubation with extracellular-vesicle-depleted CPC growth media or extracellular vesicles, 10 µL of WST reagent was added to each well. The 96-well plate underwent incubation for another 4 h, protected from light. The plate was placed on a shaker (Benchmark Scientific, Sayreville, NJ, USA) for 3 min and absorbance was measured with a BIO-TEK uQuant microplate spectrophotometer (BioTek, Winooski, VT, USA) using a reference wavelength set at 630 nm and a primary wavelength of 450 nm. The reaction was stopped with 10 µL of the stop solution. Four technical replicates were used to determine statistical significance. 

### 4.11. Statistical Analysis

Data were analyzed using Microsoft Excel and PRISM software programs and was reported as the mean +/− standard error. Relative gene expression was calculated using the 2^−ΔΔCT^ method [60]. The values were further assessed for normality using a Shapiro–Wilk test and if the values passed the normality test, an unpaired *t*-test was conducted for statistical significance against the control population. If non-normality was observed in any dataset, Wilcoxon’s *t*-test was performed to analyze statistical significance. *p* values < 0.05 were considered to be statistically significant. Actin or GAPDH was used to normalize the expression of genes and proteins of interest. 

## 5. Conclusions

Our findings provide insight into the novel transcriptome of neonatal cardiovascular-progenitor-cell-derived extracellular vesicles. We demonstrate the enhanced proliferative ability of adult cardiovascular progenitor cells upon co-culture with extracellular vesicles derived from neonatal CPCs in vitro. The neonatal cardiovascular progenitor cells contain miRNAs and transcripts that induce YAP1 transcription and activate the cell cycle in adult CPCs. This was assessed by the observation of (1) elevated levels of YAP1 transcript and protein levels; (2) elevated levels of transcripts associated with PI3K-AKT signaling such as PI3K and RelA and transcripts that function in cell cycle progression and proliferation, such as c-MYC and CCND1; (3) the elevation of transcripts involved in G1-S and G2 m progression in the cell cycle, such as CCNA2; and (4) increased proliferation in vitro. YAP1 activation and transcriptional alteration in adult CPCs by neonatal cardiovascular-progenitor-cell-derived extracellular vesicles could have a clinical benefit in cardiovascular repair through the activation of signaling in recipient cells and potentially in the surrounding cell types when applied as a cell-based treatment. 

## Figures and Tables

**Table 1:** Primer Sequences Used for RT-qPCR; (5’ to 3’ from left to right).

Human Primers	Sequence
bACTIN-FWD	TTT GAA TGA TGA GCC TTC GTC CCC
bACTIN-REV	GGT CTC AAG TCA GTG TAC AGG TAA GC
CCNA2-FWD	AGG AAA GCT TCA GCT TGT GG
CCNA2-REV	TTG AGG TAT GGG TCA GCA TC
CCND1-FWD	TTC ACA GAG CGC CAG CCA GC
CCND1-REV	CTT GGG AGC GGC GGC AAG AA
CREB – FWD	AGG TGT AGT TTG ACG CGG T
CREB-REV	GGA CTT GAA CTG TCT GCC CA
CTGF-FWD	CAC CCG GGT TAC CAA TGA CA
CTGF-REV	TCC GGG ACA GTT GTA ATG GC
ERBB4-FWD	TTC AGG ATG TGG ACG TTG CC
ERBB4-REV	GGG CAA ATG TCA GTG CAA GG
MYC – FWD	AAG ACA GCG GCA GCC CGA AC
MYC – REV	TGG GCG AGC TGC TGT CGT TG
PIK3CA-FWD	AAC AAT GCC TCC ACG ACC AT
PIK3CA-REV	TCA CGG TTG CCT ACT GGT TC
RelA-FWD	GCG AGA GGA GCA CAG ATA CC
RelA-REV	GGG GTT GTT GTT GGT CTG GA
SOX2-FWD	AAC CAG CGC ATG GAC AGT TA
SOX2-REV	GAC TTG ACC ACC GAA CCC AT
YAP1-FWD	TCC CAG ATG AAC GTC ACA GC
YAP1-REV	TCA TGG CAA AAC GAG GGT CA
KI67 – FWD	TGG CAC AAA ATA CCA TTT CCG T
KI67-REV	AGC CAA AAG TGT ACA CAG GTC A

**Table 2:** Antibodies Used for Western Blot.

Antibody	Species	Antibody Dilution	Size (kDa)	Sample Used	Catalog No.	Manufacturer
YAP1	Rabbit	1:200	65-78	0.4 mg/μL	D8H1X	Cell Signaling Technology
Beta-Actin	Mouse	1:50	45	0.4 mg/μL	8H10D10	Cell Signaling Technology

## Data Availability

The data presented in this study are openly available in FigShare: 10.6084/m9.figshare.21663674. Additional data can be made available upon reasonable request to the corresponding author.

## Supplementary Materials

The following supporting information can be downloaded at: https://www.mdpi.com/article/10.3390/ijms24098088/s1.
