# Supplementary material for: Neonatal Cardiovascular-Progenitor-Cell-Derived Extracellular Vesicles Activate YAP1 in Adult Cardiac Progenitor Cells
Source: Int J Mol Sci. 2023 Apr 30;24(9):8088. doi: 10.3390/ijms24098088 (PMC10179407; doi:10.3390/ijms24098088)
Supplement: Supplementary file 1 [file ijms-24-08088-s001.zip › ijms-2325529-supplementary.pdf]

International Journal of
Molecular Sciences

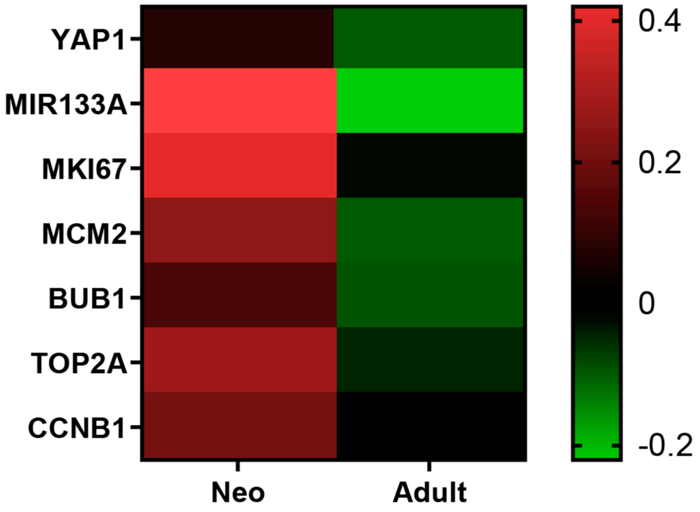

Supplementary Figure S1. Neonatal Islet-1+ CPC derived extracellular vesicles carry YAP1 and are enriched with transcripts that promote proliferation.
